# *Spondias tuberosa* Seed as a Source of Bioactives by an Optimized Microwave-Assisted Green Extraction

**DOI:** 10.1021/acsomega.5c08226

**Published:** 2025-11-25

**Authors:** Ester Fonseca da Conceição, Carolline Margot Albanez Lorentino, Thayssa da Silva Ferreira Fagundes, Alex de Aguiar Novo, Claudete Norie Kunigami, André Luis Souza dos Santos, Davyson de Lima Moreira, Eliane Przytyk Jung, Leilson de Oliveira Ribeiro

**Affiliations:** † Laboratory of Organic and Inorganic Chemical Analysis, National Institute of Technology, Av. Venezuela, 82, Saúde, Rio de Janeiro, Rio de Janeiro 20081-312, Brazil; ‡ Laboratory for Advanced Studies of Emerging and Resistant Microorganisms, Microbiology Institute Paulo de Góes, Federal University of Rio de Janeiro, Av. Carlos Chagas Filho, 373 - Cidade Universitária, Rio de Janeiro, Rio de Janeiro 21941-599, Brazil; § Laboratory of Natural Products, Rio de Janeiro Botanical Garden Research Institute, Rua Pacheco Leão, 915 - Jardim Botânico, Rio de Janeiro, Rio de Janeiro 22460-030, Brazil; ⊥ Plant Biotechnology Center, Roberto Alcantara Gomes Institute of Biology, Rio de Janeiro State University, Rua São Francisco Xavier, 524, Haroldo Lisboa da Cunha Pavilion-505, Maracanã, Rio de Janeiro 20550-013, Brazil

## Abstract

Umbu (*Spondias tuberosa*Arruda) is a native Brazilian Caatinga fruit. The pulp is the main product of its agroindustrialization; however, depulping generates approximately 25% of residue. This study aimed to recover bioactive compounds from umbu seed through a more sustainable approach, using propylene glycol as a solvent in microwave-assisted solid–liquid extraction. Additionally, the study evaluated the chemical profile, photoprotective action, and *in vitro* toxicity. An experimental design using percentage of propylene glycol (15–85%) and temperature (59–201 °C) as independent variables was adopted. Total phenolic content (TPC) and antioxidant capacity by DPPH^•^, ABTS^•+^, and FRAP were evaluated as responses. The highest response values were observed at 201 °C and 60% propylene glycol. Adjusting the extraction time to 15 min resulted in a 41% increase in TPC. According to UPLC-HRMS/MS analysis, the optimized extract is mainly composed of phenolic compounds. It showed a sun protection factor of 13 at 30 mg/mL. Furthermore, the 25% extract showed no toxicity in the *G. mellonella* model. Therefore, the use of propylene glycol as a green solvent in microwave-assisted extraction favored the recovery of bioactive compounds from umbu seed, enabling the production of a bioproduct with potential cosmetic application.

## Graphical abstract

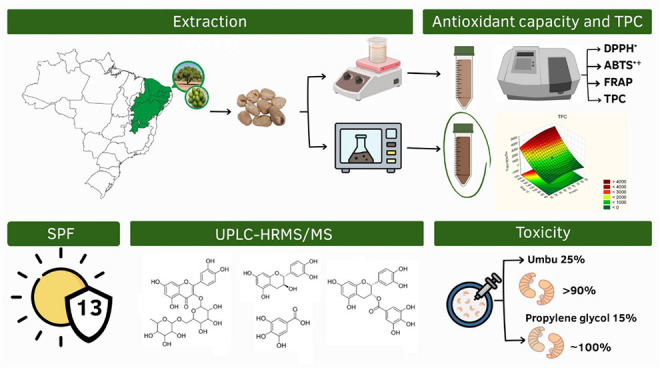

## Introduction

A large amount of solid and liquid food waste is generated worldwide, representing a significant environmental issue. This waste originates from various stages of the food supply chain including industrial food processing, quality deterioration during retail handling, food service operations, and household consumption. However, byproducts such as fruit peels, seeds, stems, and processed vegetable residues are rich in bioactive compounds, including phenolics, vitamins, carotenoids, and alkaloids.^1^

Bioactive compounds derived from plants and fruits have attracted considerable research interest due to their technological potential and health benefits.^2^ Among these are phenolic compounds, which are widely distributed in the plant kingdom and can be found in fruit peels and seeds. They are beneficial for human health due to their high antioxidant potential, neutralizing the formation of free radicals involved in oxidation processes. Studies link the consumption of these substances against the effects of age-related disorders and diseases such as cancer, diabetes, and hypertension. They also play a significant role in the cosmetic industry as natural antioxidants and photoprotective agents.^3–5^

In this context, the rising demand for natural ingredients across multiple sectors has been a key driver of growth in the plant extract industry. Valued at 36.7 billion USD in 2023, this global market is projected to expand at a compound annual growth rate (CAGR) exceeding 11.3% between 2024 and 2032. These figures underscore the scientific and industrial relevance of research aimed at developing plant extracts from waste materials for sustainable applications.^6^

Umbu (*Spondias tuberosa* Arruda) is a fruit tree native to the Brazilian Caatinga and belongs to the Anacardiaceae family. Many bioactive compounds have been described in its phytochemical composition, such as quercetin, rutin, and vitamin C. Furthermore, the fruit presents a relevant socioeconomic importance since its cultivation can increase the income of families from the semiarid region of Brazil.^7^ The frozen pulp is the main product obtained from umbu fruit. However, the fruit depulping process can generate a total of 25% waste.^8^ This waste is composed of seed, peel, and refining cake, which still contains valuable compounds.^7^ It was reported by Freitas et al.^9^ that umbu seeds are rich in dietary fiber and phenolic compounds. Studies done by Dias et al. showed the presence of palmitic, stearic, oleic, linoleic, and linolenic acids in the lipidic fraction analysis of umbu seed extracts.^10^

The conventional extraction methodologies for bioactive compounds from plant waste predominantly utilize conventional organic solvents, including methanol, acetone, hexane, and ethyl acetate. However, they present some disadvantages as they are dangerous, toxic, flammable, of low or nonselectivity, harmful to the environment, and generally derived from nonrenewable sources.^11^ This makes the procedure dangerous and requires purification steps before extract application. Thus, to avoid these issues and make the process more sustainable, safer, and more environmentally friendly, green solvents have been evaluated as ethanol, deep eutectic solvents, and vegetable oil, for example.^12^

Propylene glycol is a clear, slightly viscous, and water-miscible liquid. These characteristics make it a promising alternative to conventional organic solvents, which are often flammable and toxic. Because it is nontoxic, a bioactive propylene glycol extract can be used directly in formulations. Based on these properties, it has been used as a solvent for bioactive compounds, aromas, essences, and fragrances.^13^

For example, propylene glycol was used as a solvent in the recovery of bioactive compounds from coffee pulp,^14^ in which ultrasound-assisted extraction showed significantly higher values of total phenolic and antioxidant capacity than those employing ultrasound-assisted extraction with ethanol as the solvent. In the extraction of *Terminalia chebula* Retz,^15^ it was noted that water-propylene glycol extracts showed higher antioxidant activity than those of water-ethanol extracts.

Conventional solid–liquid extraction of bioactive compounds from plants depends on contacting the sample with the appropriate solvents for a certain time. Soxhlet, maceration, and hydrodistillation are the most employed techniques, which require large amounts of solvent and long extraction times. In order to increase recovery efficiency, reduce processing time, and solvent consumption, nonconventional extraction techniques such as enzyme-assisted extraction, ultrasound-assisted extraction, high hydrostatic pressure-assisted extraction, microwave-assisted extraction (MAE), and electric field have been evaluated. Many studies have demonstrated superior results from these techniques when compared to those conventional extractions.^16^

Among thermal extraction techniques, MAE provides high extraction efficiency when compared to conventional heat in the shortest processing times. However, the power applied to MAE should be carefully optimized to avoid the degradation of thermally sensitive compounds when subjected to high temperatures. On the other hand, MAE is considered a perfectly scalable technique to be implemented at the pilot or industrial plant level. MAE is based on the principle of electromagnetic irradiation of the polar solvent and the sample, resulting in superheating the plant matrix material and its rupture. The fast heating is obtained by ionic conduction and dipole rotation mechanisms.^17^ Results reported by Dairi et al.,^18^ Sharma et al.,^19^ and Buratto et al.^20^ showed that MAE increased phenolic concentration in extracts of red onion, sea buckthorn pomace, and açaí pulp in comparison with conventional techniques. In addition, when compared with conventional heating methods, it exhibits a shorter processing time as it is possible to reach high temperatures in a short time as the heat is dissipated evenly in the plant material, reducing the degradation of target compounds.^21^

Considering the above and given that microwave-assisted extraction using propylene glycol as a solvent has not yet been explored for obtaining bioactive compounds from umbu seed, this study aimed to optimize the extraction conditions and characterize the resulting bioactive compounds. Extraction efficiency was evaluated based on total phenolic content and antioxidant capacity. Additionally, the optimized extract was further analyzed for its metabolomics profile by UPLC-HRMS/MS, sun protection factor (SPF), and *in vivo* toxicity.

## Results and Discussion

### Extraction Evaluation

Eleven extractions were carried out, ranging the temperature from 59 to 201 °C and using different binary mixtures of propylene glycol and distilled water as solvent (15%–85%) for 10 min. As presented in Table 1, both propylene glycol percentage and processing temperature had a significant impact on the evaluated parameters, as demonstrated by the marked variations in TPC values and antioxidant capacity, assessed through different analytical methods. The highest TPC value (assay 8:50% propylene glycol, 201 °C – 3181 mg GAE/100 g) was 8-fold higher than that obtained in the worst experimental condition (assay 1:25% propylene glycol, 80 °C – 410 mg GAE/100 g). It is worth highlighting that assay 8 showed higher TPC values than the extract of umbu seed obtained by conventional extraction using acetone as solvent (947 mg GAE/100g).^22^ Moreover, our result was higher than that reported by Ribeiro et al.^7^ who extracted phenolic compounds of umbu seed using 70% acetone by conventional extraction (∼1200 mg GAE/100 g) and by Freitas et al.^9^ who extracted phenolics of umbu seed at different maturation stages using an ultrasound bath and ethanol:water as solvent (194–276 mg GAE/100 g).

**Table 1:** Experimental Design for Extraction and Results for Total Phenolic Content (TPC) and Antioxidant Capacity by DPPH^•^, ABTS^•+^, and FRAP Assays^a^

Propylene glycol (%)	Temperature (°C)	DPPH^•^ (μmol Trolox/g)	TPC (mg GAE/100 g)	ABTS^•+^ (μmol Trolox/g)	FRAP (μmol Fe^2+^/g)
25	80	16	410	23	71
25	180	99	2311	85	300
75	80	21	756	39	86
75	180	143	3179	153	339
15	130	22	960	44	135
85	130	56	1159	46	126
50	59	20	561	30	85
50	201	162	3181	190	442
50	130	71	1659	72	185
50	130	65	1468	72	178
50	130	64	1426	67	154

a GAE – gallic acid equivalent.

The assessment of the antioxidant capacity of the extracts using the DPPH^•^ assay revealed values ranging from 16 to 162 μmol of Trolox/g. Notably, the highest antioxidant capacity was observed in assay 8 conducted at 201 °C with 50% propylene glycol, exhibiting a 10-fold increase compared to the other samples. The same pattern was observed for the antioxidant capacity determined via the FRAP assay. In this condition, the result was 442 μmol Fe^2+^/g. For the results of antioxidant capacity via the ABTS^•+^ assay, values ranged from 23 to 190 μmol Trolox/g, with the best response being 8-fold higher than that obtained in the worst condition (assay 1:25% propylene glycol, 80 °C). Thus, a strong correlation between antioxidant capacities and TPC values was observed, which was confirmed by Pearson’s correlation analysis (*r* > 0.96, *p* < 0.05). It is relevant to note that the antioxidant capacity obtained under the best experimental condition in the current work is much higher than that reported by Freitas et al.,^22^ who evaluated the recovery of antioxidant compounds from umbu seed using 70% acetone as a solvent in a conventional extraction, conducted in 30 min at 60 °C (44 μmol of Trolox/g).

Pareto charts (Figures 1–4) indicate that both the temperature and solvent composition significantly influenced the extraction efficiency of bioactive compounds. Among the evaluated variables, temperature exerted the most pronounced effect on extraction, particularly for the FRAP assay. The linear effect of temperature was positive (*p* < 0.05), indicating that higher temperatures enhance the recovery of bioactive compounds from umbu seed. However, the quadratic effect of temperature was also significant, suggesting the existence of an optimal temperature range beyond which the efficiency may decline. Elevated temperatures facilitate the extraction of bioactive compounds by reducing solvent viscosity, thereby improving solvent–sample interactions, increasing compound solubility, and enhancing the diffusion coefficient.^8^ Nonetheless, excessively high temperatures can lead to the thermal degradation of certain compounds, reducing overall extraction efficiency and underscoring the need for process optimization.^13^ Jaouhari et al.,^23^ when recovering phenolic compounds from raspberry pomace by varying the temperature from 100 to 200 °C and time from 1 to 40 min, also reported better results for total phenolics and total flavonoids at the highest temperature at a short time (10 min), corroborating the thermal stability of these compounds. In the optimized extract, gallic acid, protocatechuic acid, 4-hydroxybenzoic acid, and flavonoids such as catechin, rutin, epicatechin, and vanillin, among others, were identified.

**Figure 1:**
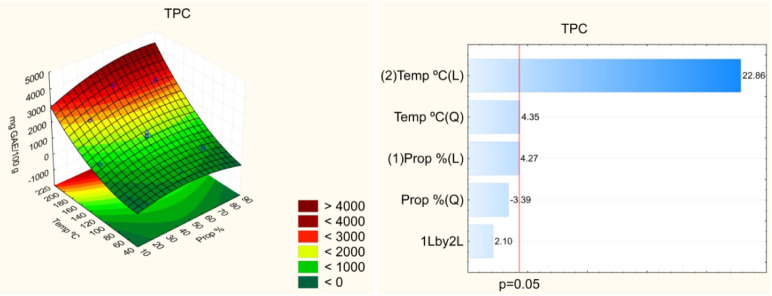
Effect of the independent variables on the total phenolic content (TPC) and response surface.

**2:**
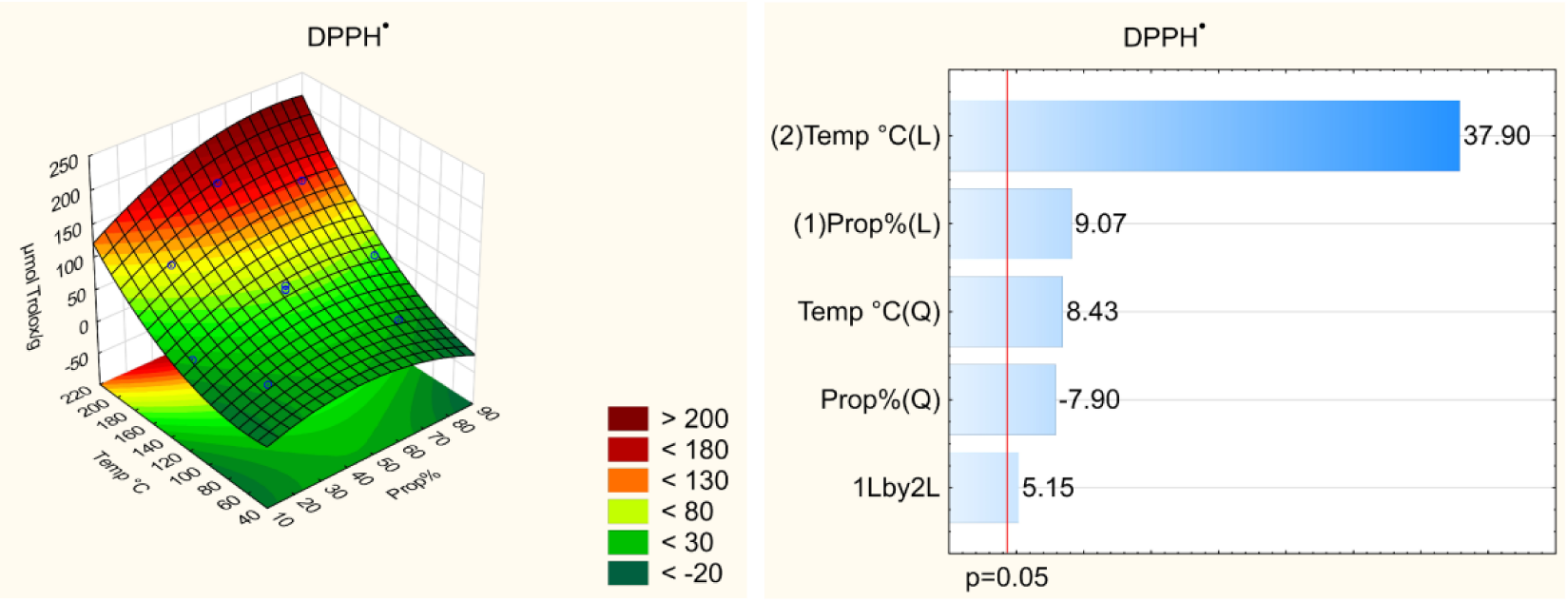
Effect of the independent variables on the antioxidant capacity by the DPPH^•^ assay and response surface.

**3:**
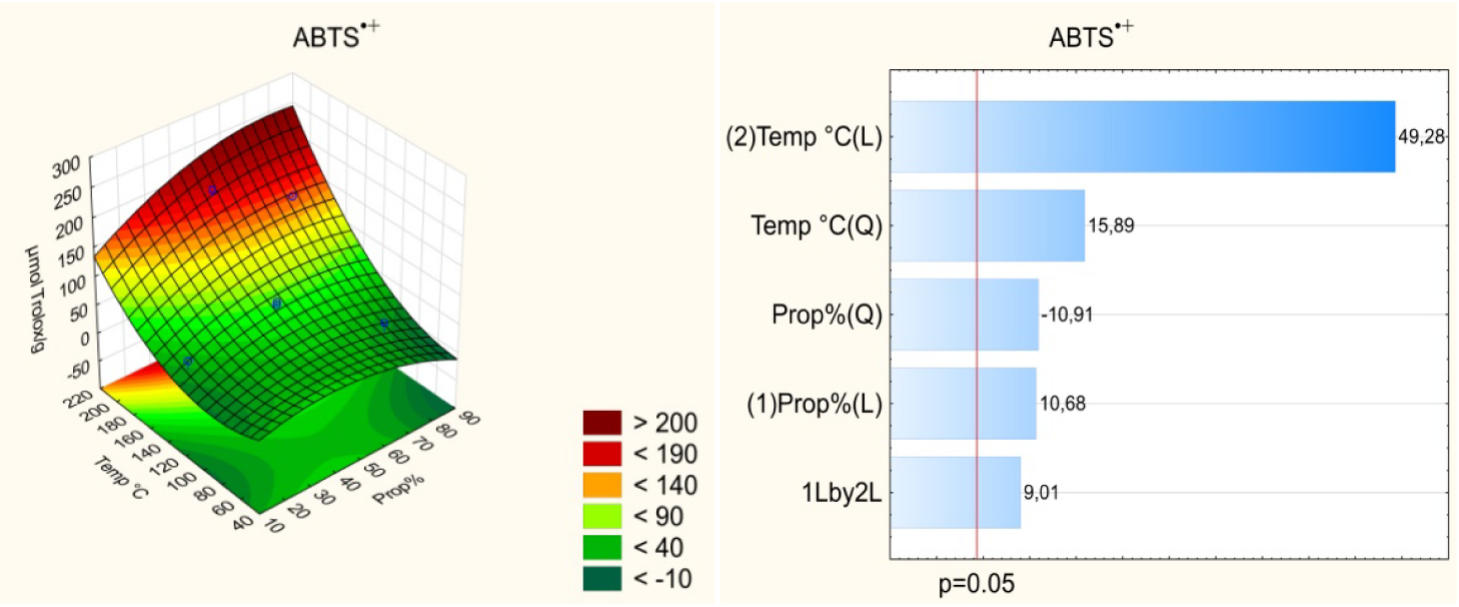
Effect of the independent variables on the antioxidant capacity by the ABTS^•+^ assay and response surface.

**Figure 4:**
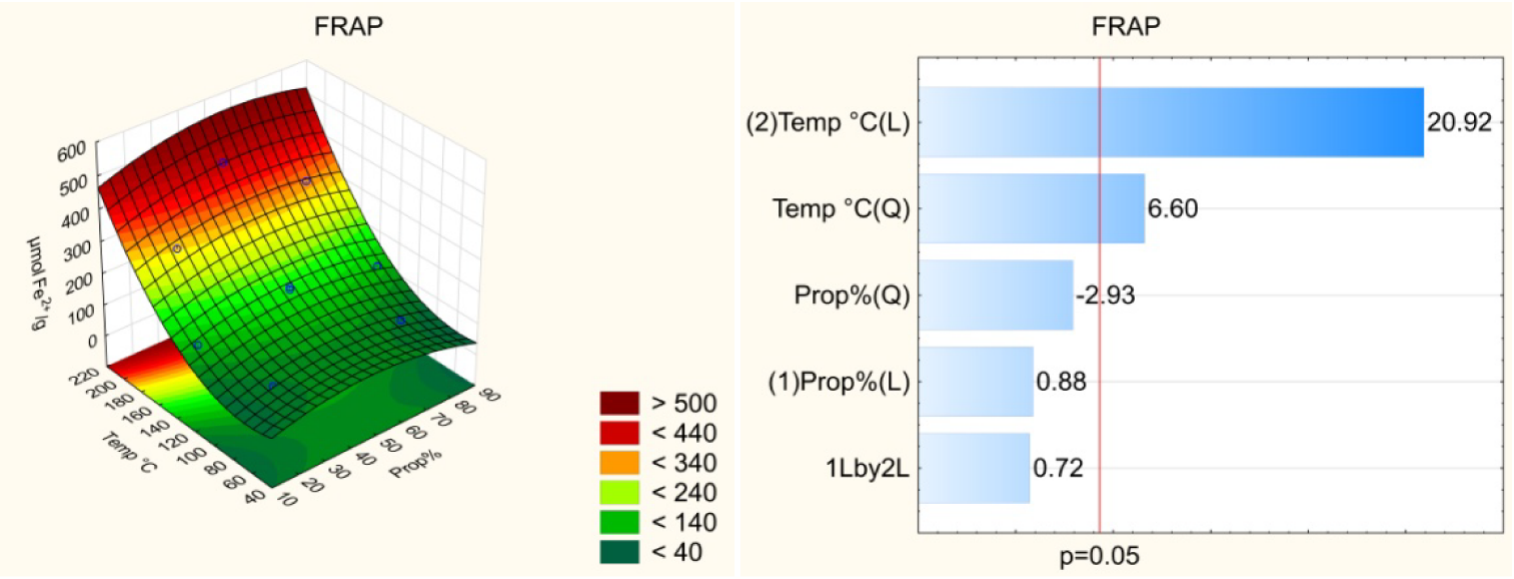
Effect of the independent variables on the antioxidant capacity by the FRAP assay and response surface.

The positive linear effect of propylene glycol concentration in the solvent was also significant, indicating that increasing the proportion of propylene glycol improved the extraction efficiency. This pattern can be attributed to the chemical affinity of propylene glycol with various bioactive compounds; as a solvent of intermediate polarity, it interacts effectively with a broad range of compounds, including phenolics. Moreover, combining propylene glycol with water is essential to reduce the overall viscosity of the solvent system as propylene glycol is moderately viscous. Water also enhances the solubilization of more polar bioactive compounds Figure 4.^11^

The statistical analysis of the data shows that all models were important for predicting the pattern of the responses since the values of *F* calculated (33, 295, 23, and 121 for TPC, DPPH^•^, ABTS^•+^, and FRAP, respectively) were higher than the *F* value listed (*F*_5.5_ = 5.05) at α = 0.05. The only models that lack fit were ABTS^•+^ and TPC. The adjusted *R* ^2^ values exceeded 0.92, indicating that the model explains more than 92% of the variability in the data and demonstrates an excellent goodness-of-fit.

### Optimization

Optimization was performed using a multiple-response methodology based on the desirability function, which determines optimal conditions by calculating the geometric mean of the experimental responses. The approach produces both individual and overall desirability profiles, with overall desirability values ranging from 0 to 1, where values approaching 1 denote superior process optimization. In this study, relative importance levels of the responses were set to 1, considering that they have the same importance in the process of extraction optimization. As shown in Figure 5, the optimal conditions were identified as a solvent composition of 60% propylene glycol in water and an extraction temperature of 201 °C, achieving an overall desirability (*D*) of 0.9999. Propylene glycol percentages above 60% did not improve the extraction of antioxidant compounds; therefore, 60% propylene glycol was selected to minimize solvent costs. Extraction temperature showed a positive effect on the responses, but values above 201 °C were not evaluated due to equipment limitations. Higher temperatures would also increase the process costs through greater energy demand. In addition, it is important to emphasize that the *D* value was 0.9999, corroborating that this is the optimal condition to obtain an antioxidant extract from umbu seed.

**Figure 5:**
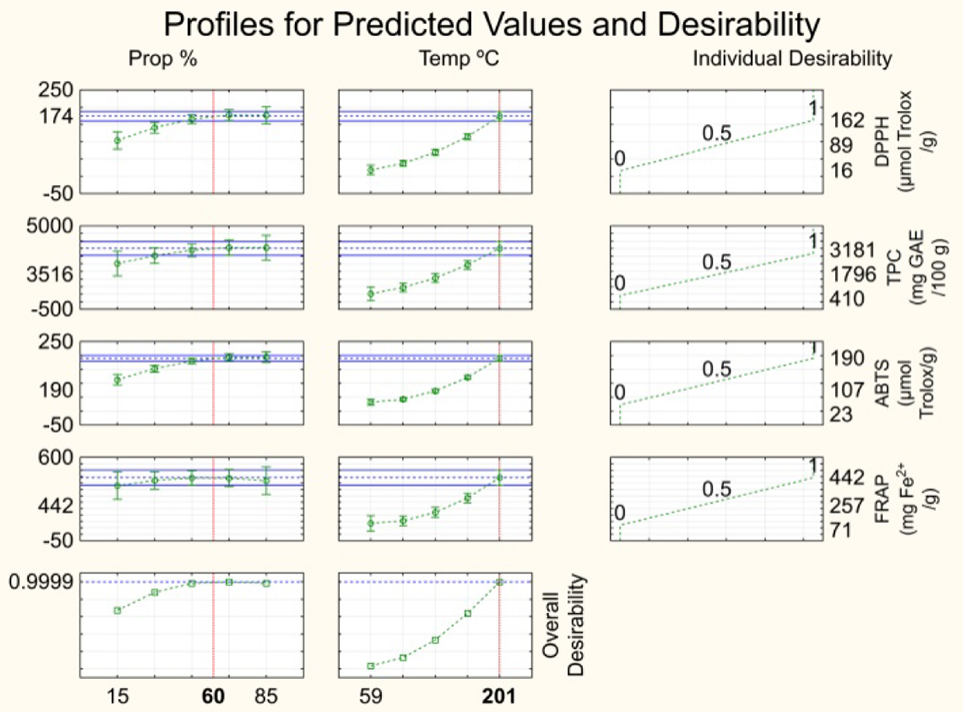
Desirability function is used to select the optimal operational condition for extraction.

Considering the results shown in Table 2, it was possible to register that the TPC value and antioxidant capacity of the extracts, obtained under the condition recommended by the desirability function, presented values close to those predicted by the mathematical model, while relative standard deviation (RSD) was lower than 9%. Therefore, the optimized condition was validated. Also, these values are in a 95% confidence interval.

**Table 2:** Predicted and Observed Results for Total Phenolic Content (TPC) and Antioxidant Capacity of the Umbu Seed Extract Obtained under Optimal Condition^a^

	DPPH^•^ (μmol Trolox/g)	TPC (mg GAE/100 g)	ABTS^•+^ (μmol Trolox/g)	FRAP (μmol Fe^2+^/g)
Observed results	183.12 ± 6.90	3,985.16 ± 157.47	192.13 ± 13.22	501.39 ± 17.99
Predicted results	174.00	3,516.00	190.00	442.00
RSD (%)	3.61	8.84	0.79	8.90

a GAE – gallic acid equivalent. RSD = relative standard deviation.

### Extraction Kinetics

In the study of extraction kinetics, the aim was to evaluate the influence of time on the recovery of bioactive compounds from the umbu seed. Therefore, the time after reaching a temperature of 201 °C varied from 0 to 20 min, keeping the solvent composition 60% propylene glycol fixed in accordance with the validated optimal condition.

Figures 6 and 7 show the kinetic profiles for TPC and antioxidant capacity. Although the antioxidant capacity values for intervals between 10 and 20 min were statistically not significant, except for the FRAP assay (the highest value found at 20 min), the result for TPC was considerably higher in 15 min of extraction, with a 41% increase in relation to the fixed condition in the previous step (3,985.16 mg GAE/100 g). Furthermore, above 15 min of extraction, there was a relevant loss of these compounds, as shown in Figure 7.

**Figure 6:**
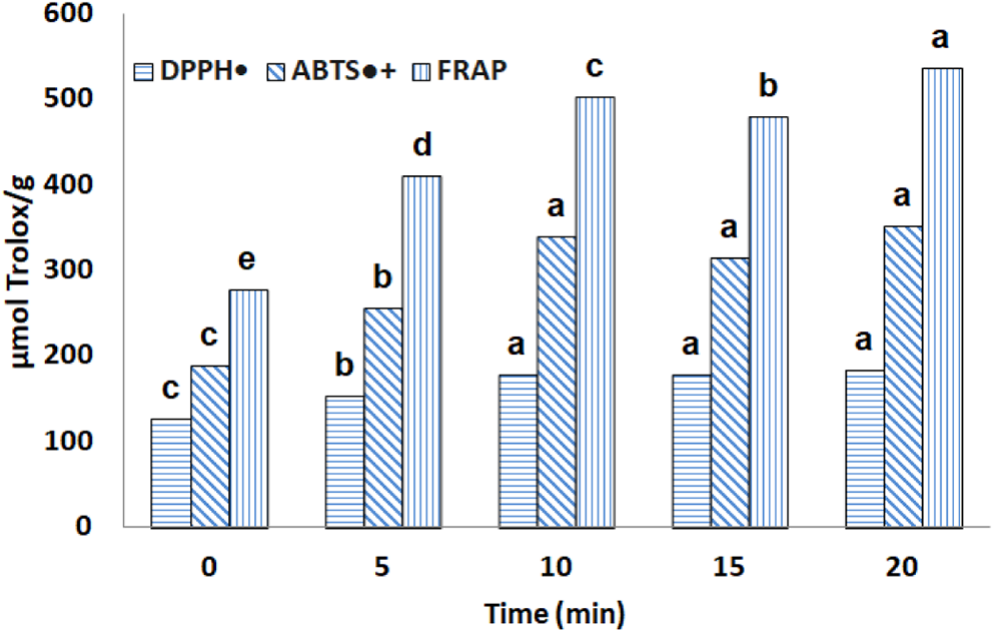
Influence of the extraction time on the antioxidant capacity of the umbu seed extract. Bars with the same letters show that the results are equal to each other (*p* > 0.05).

**Figure 7:**
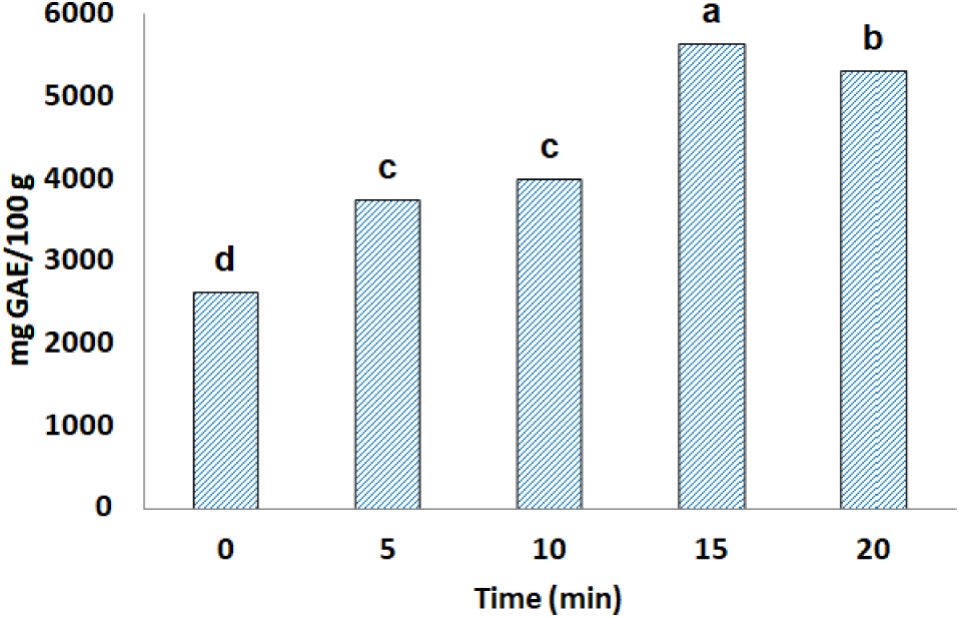
Influence of the extraction time on the total phenolic content (TPC) of the umbu seed extract. Bars with the same letters show that the results are equal to each other (*p* > 0.05).

This pattern can be explained by the fact that in a longer time, the solvent is in contact with the sample; the higher their interaction, the better the extraction of bioactive compounds. However, prolonged exposure of the sample to the high temperature of the medium may lead to degradation of the desired compounds and then compromising extraction. Therefore, to achieve the highest efficiency in the shortest time, with the aim of saving both time and energy, 15 min was chosen as the ideal time for extraction of antioxidant compounds from umbu seed.

### Conventional and Microwave-Assisted Solid–Liquid Extraction

The extraction of bioactive compounds by conventional solid–liquid extraction was performed with the aim of comparing its efficiency with that of microwave-assisted solid–liquid extraction using 60% propylene glycol as the solvent. The results of the analysis of antioxidant capacity and TPC are presented in Table 3. It can be observed that the extraction time that generated the highest responses in most analyses was 60 min, except for the ABTS^•+^ assay. Nonetheless, despite the highest values for this technique, it remains considerably lower than those obtained under the optimized microwave-assisted extraction conditions (Table 4). The result of the TPC for optimal microwave-assisted extraction (5,638.1 mg of GAE/100 g) is 8-fold higher than that for conventional extraction (721.85 mg of GAE/100 g), and the antioxidant capacity using the DPPH^•^ assay is 3-fold higher (178.53 μmol of Trolox/g and 53.67 μmol of Trolox/g, respectively). The TPC value obtained in the present study for conventional extraction was lower than those reported by Freitas et al.^22^ and Ribeiro et al.,^7^ who employed acetone as the solvent in the conventional extraction of phenolics from umbu seed (947 mg GAE/100 g and ∼1200 mg GAE/100 g, respectively). This difference can be mainly attributed to solvent viscosity, as 60% propylene glycol was used in the present study. Therefore, it is concluded that extracting the bioactive compounds of umbu seed by microwave-assisted extraction is more efficient than conventional heating when propylene glycol is used as a solvent.

**Table 3:** Results for Total Phenolic Content (TPC) and Antioxidant Capacity of the Umbu Seed Extract Obtained by Conventional Solid–Liquid Extraction^a^

Time (min)	DPPH^•^ (μmol Trolox/g)	TPC (mg GAE/100 g)	ABTS^•+^ (μmol Trolox/g)	FRAP (μmol Fe^2+^/g)
15	44.34 ± 1.17^c^	574.5 ± 6.73^d^	54.98 ± 1.53^b^	100.79± 2.03^c^
30	48.62 ± 0.86^b^	670.66 ± 10.79^b,c^	60.87 ± 1.30^a^	113.63 ± 0.93^b^
60	53.67 ± 0.68^a^	721.85 ± 7.53^a^	46.33 ± 1.09^c^	126.92 ± 3.39^a^
90	44.48 ± 1.62^c^	647.69 ± 11.61^c^	47.04 ± 1.22^c^	97.66 ± 0.98^c^
120	44.94 ± 0.80^c^	678.84 ± 25.71^b^	43.40 ± 0.99^d^	87.30 ± 1.34^d^

a GAE – gallic acid equivalent. Same letters in the same column show that the results are not statistically significant (*p* > 0.05).

**Table 4:** Results for Total Phenolic Content (TPC) and Antioxidant Capacity of the Umbu Seed Extract Obtained by Conventional Solid–Liquid Extraction (CSLE) and Microwave-Assisted Solid–Liquid Extraction (MASLE)^a^

Techniques	DPPH^•^ (μmol Trolox/g)	TPC (mg GAE/100 g)	ABTS^•+^ (μmol Trolox/g)	FRAP (μmol Fe^2+^/g)
CSLE	53.67 ± 0.68^b^	721.85 ± 7.53^b^	46.33 ± 1.09^b^	126.92 ± 3.39^b^
MASLE	178.53 ± 3.83^a^	5,638.1 ± 213.37^a^	315.89 ± 21.17^a^	479.46 ± 3.68^a^

a GAE – gallic acid equivalent. Same letters in the same column show that the results are not statistically significant (*p* > 0.05).

It is also important to note that microwave-assisted extraction of bioactive compounds from plants is scalable, further underscoring the relevance of studies employing this technique. Oke et al.^24^ applied microwave-assisted extraction for the recovery of bioactive compounds from *Hunteria umbellata* seeds. In addition to process optimization (extraction time of 2 min, microwave power of 780 W, and solid–liquid ratio of 0.4 g/mL), their study included a techno-economic scale-up with sensitivity analysis. The results demonstrated the feasibility of the process at a larger scale, with a batch size of 5 kg, a batch time of 137 min, a production rate of 0.036 kg/min, a total capital investment of USD 80,398, an annual production cost of USD 456,000, and a payback period of 2.29 years.

### Chemical Analysis

The crude extract (UMBU B) and the purified extract subjected to SPE (UMBU P) were analyzed using ultraperformance liquid chromatography coupled to high-resolution mass spectrometry in tandem (UPLC-HRMS/MS), in positive and negative ionization modes by electrospray ionization (ESI). Chromatograms can be seen in Figures S1 and S2.

The data were processed by using the GNPS platform to generate molecular networks and annotate the compounds. The base peak chromatograms obtained from the SPE-treated sample (UMBU P) in both ionization modes showed higher intensity and a greater variety of detected ions, indicating that the cleanup step was effective in facilitating the compound ionization.

The molecular networks showed a total of **708** nodes in the negative ionization mode and **234** nodes in the positive ionization mode after removing the ions from the blank sample analysis. **Eight** compound clusters with similar mass spectra, also known as molecular families, were identified, including phenolic acids, catechin gallates, catechins, flavonoids, lignans, glycerides, glycerophospholipids, phenylpropanoids, and stilbenoids. For more details, see Figures S3–S9. In total, **46** compounds were annotated in the molecular networks using the GNPS spectral library and manual data inspection. Figure 8 shows the 8 annotated molecular families, highlighting one representative compound from each cluster. The list of the total annotated compounds (1–46) can be found in Table 5.

**Figure 8:**
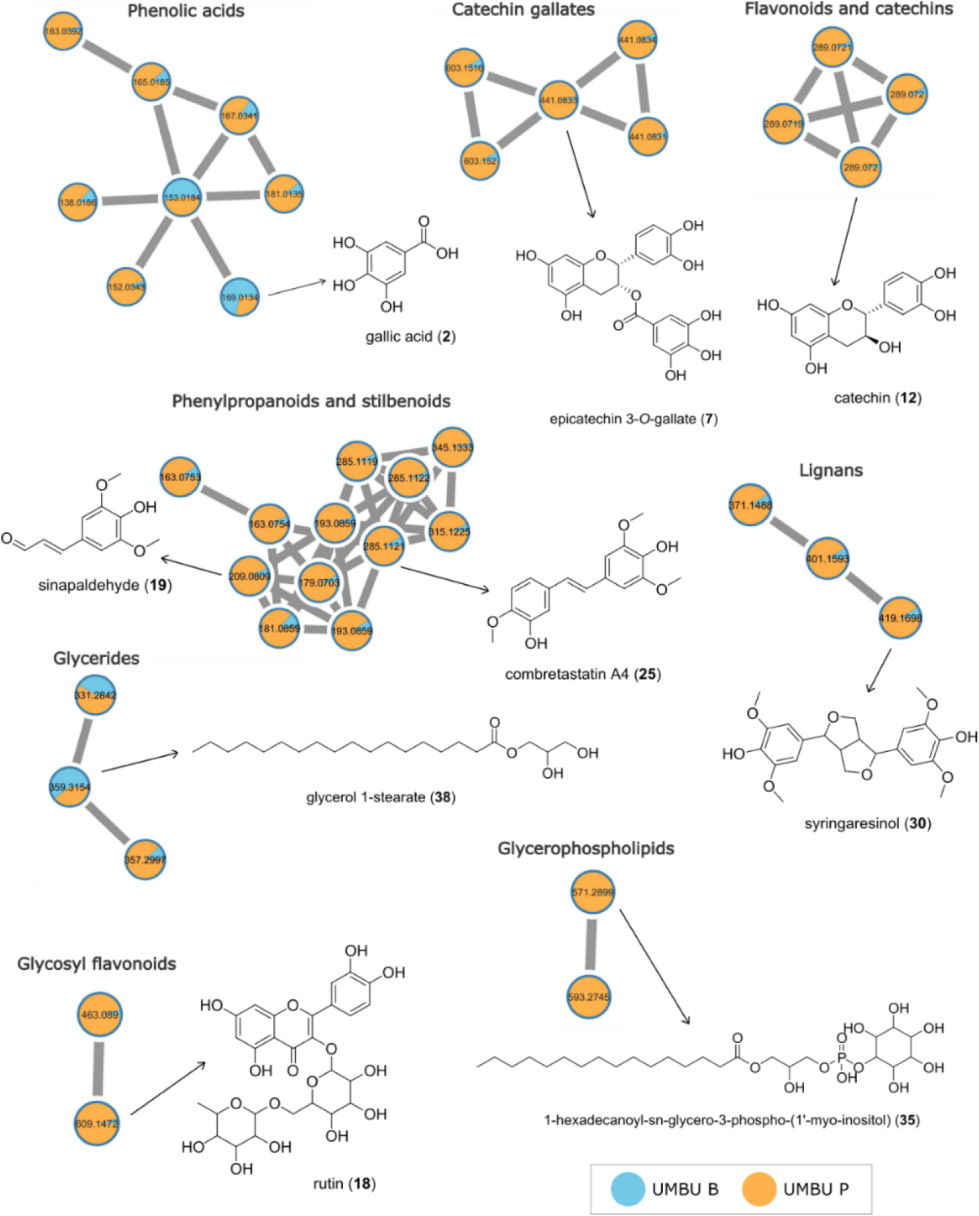
Molecular families of the compounds in both positive and negative ionization modes with a representative entity from each chemical class. Annotated compounds with no grouping with other ions (self-loops) are not shown here. The node colors indicate the relative abundance of compounds in each extract: blue represents the crude extract (UMBU B), and orange represents the extract subjected to cleanup in SPE (UMBU P).

**Table 5:** Ultra-Performance Liquid Chromatography Coupled to High-Resolution Mass Spectrometry in Tandem (UPLC-HRMS/MS) Data for the Chemical Composition of Umbu Seed Extracts

Compound number	Compound name	Molecular formula	Rt (min)	Adduct ion	Experimental *m*/*z*	Theoretical *m*/*z*	Error (ppm)	Main ions of MS^2^ spectrum
1	vanillic acid	C_8_H_8_O_4_	0.64	[M – H]^−^	167.0341	167.0344	–2.0	167, 149, 139, 123, 109, 95
2	gallic acid	C_7_H_6_O_5_	0.45	[M – H]^−^	169.0134	169.0137	–1.7	169, 141, 125,123, 101, 99, 97
3	protocatechuic acid	C_7_H_6_O_4_	0.77	[M – H]^−^	153.0184	153.0188	–2.5	153, 109, 81, 64
4	terephthalic acid^a^	C_8_H_6_O_4_	1.46	[M – H]^−^	165.0185	165.0188	–1.7	165, 150, 137, 121, 97
5	4-acetylbenzoic acid	C_9_H_8_O_3_	2.74	[M – H]^−^	163.0392	163.0395	–1.9	163, 119, 93
6	7-hydroxy-1,3-benzodioxole-5-carboxylic acid	C_8_H_6_O_5_	0.71	[M – H]^−^	181.0135	181.0137	–1.0	181, 166, 137, 109, 96
7	epicatechin-3-*O*-gallate	C_22_H_18_O_10_	4.81	[M – H]^−^	441.0833	441.0822	2.6	441, 289, 245, 169, 125
8	epicatechin-5-*O*-gallate	C_22_H_18_O_10_	5.01	[M – H]^−^	441.0831	441.0822	2.1	289, 245, 169, 137, 125
9	epicatechin-7-*O*-gallate	C_22_H_18_O_10_	5.60	[M – H]^−^	441.0834	441.0822	2.8	289, 245, 179, 137, 125
10	epicatechin-3-*O*-gallate-7-*O*-glucoside	C_28_H_28_O_15_	7.02	[M – H]^−^	603.1516	603.1350	27.5^b^	451, 433, 301, 299, 245, 169, 125, 68
11	epicatechin-3-*O*-gallate-4́-*O*-glucoside	C_28_H_28_O_15_	6,76	[M – H]^−^	603.152	603.1350	28.2^b^	451, 433, 301, 289, 271, 257, 245, 169, 125, 57
12	catechin	C_15_H_14_O_6_	0.94	[M – H]^−^	289.072	289.0712	2.7	245, 221, 203, 179, 151, 137, 125, 109
13	epicatechin	C_15_H_14_O_6_	0.66	[M – H]^−^	289.072	289.0712	2.7	245, 221, 203, 179, 151, 137, 125, 109
14	mesquitol	C_15_H_14_O_6_	1.72	[M – H]^−^	289.0721	289.0712	3.1	245, 221, 203, 179, 151, 137, 125, 109
15	iso-mesquitol	C_15_H_14_O_6_	1.34	[M – H]^−^	289.0719	289.0712	2.4	245, 221, 203, 179, 151, 137, 125, 109
16	quercetin	C_15_H_10_O_7_	6.79	[M – H]^−^	301.0357	301.0348	2.9	301, 179, 151
17	quercetin-3-glucoside	C_21_H_20_O_12_	5.69	[M – H]^−^	463.089	463.0877	2.9	300, 271, 255, 178, 151
18	rutin	C_27_H_30_O_16_	5.72	[M – H]^−^	609.1472	609.1456	2.7	300, 271, 255, 178, 151
19	sinapaldehyde	C_11_H_12_O_4_	4.77	[M + H]^+^	209.0809	209.0814	–2.3	209, 191, 181, 177, 103, 153, 149, 145, 131, 121, 103, 55
20	coniferaldehyde	C_10_H_10_O_3_	4.30	[M + H]^+^	179.0703	179.0708	–2.8	179, 161, 147, 133, 123, 119, 115, 105, 91, 55
21	sinapyl alcohol	C_11_H_14_O_4_	6.15	[M + H–H_2_O]^+^	193.0859	193.0864	–2.6	193, 178, 161, 143, 133, 115, 105, 91, 79
22	4-vinylsyringol	C_10_H_12_O_3_	6.47	[M + H]^+^	181.0859	181.0865	–3.1	181, 149, 121, 103, 93
23	coniferyl alcohol	C_10_H_12_O_3_	2.45	[M + H–H_2_O]^+^	163.0754	163.0759	–3.1	163, 131, 103,
24	5-[3-hydroxyprop-1-enyl]-2,3-dimethoxyphenol	C_11_H_14_O_4_	2.97	[M + H–H_2_O]^+^	193.0859	193.0865	–2.9	193, 178, 161, 143, 133, 115, 105, 91, 79
25	combretastatin A4	C_17_H_18_O_5_	6.96	[M + H–H_2_O]^+^	285.1121	285.1127	–2.0	285, 253, 225, 161
26	4,4’-1,2-ethenediylbis[2,6-dimethoxyphenol]	C_18_H_20_O_6_	6.47	[M + H–H_2_O]^+^	315.1225	315.1232	–2.3	315, 283, 255, 223, 161
27	4-[2-(4-hydroxy-3-methoxyphenyl)ethenyl]-2,6-dimethoxyphenol	C_17_H_18_O_5_	5.66	[M + H–H_2_O]^+^	285.1122	285.1127	–1.7	285, 253, 225
28	4-[2-(4-hydroxy-3-methoxyphenyl)ethenyl]-2,3-dimethoxyphenol	C_17_H_18_O_5_	6.52	[M + H–H_2_O]^+^	285.1119	285.1127	–2.7	285, 253, 225
29	rhapontigenin	C_15_H_14_O_4_	6.49	[M–H]^−^	257.0821	257.0814	2.8	257, 242, 241, 239, 229, 228
30	syringaresinol	C_22_H_26_O_8_	6.52	[M + H]^+^	419.1698	419.1706	–1.9	401, 383, 330, 315, 235, 217, 205, 173, 167
31	fargesin	C_21_H_22_O_6_	6.52	[M + H]^+^	371.1488	371.1495	–1.8	371, 353, 341, 321, 300, 247, 217, 167, 137, 91
32	3-methoxy-fargesin	C_22_H_24_O_7_	6.52	[M + H]^+^	401.1593	401.1600	–1.8	401, 383, 351, 343, 330, 315, 247, 217, 167, 107
33	secoisolariciresinol	C_20_H_26_O_6_	6.07	[M – H]^−^	361.1661	361.1651	2.7	346, 315, 179, 165, 122
34	lariciresinol	C_20_H_24_O_6_	6.25	[M – H]^−^	359.1505	359.1495	2.9	314, 299, 283, 193, 191, 157, 93, 89, 75, 71, 69, 61
35	1-hexadecanoyl-sn-glycero-3-phospho-(1’-myo-inositol)	C_25_H_49_O_12_P	11.05	[M – H]^−^	571.2899	571.2883	2.7	315, 255, 241, 153
36	(9,12,15)-octadecatrienoyl-glycero-3-phospho-(1’-myo-inositol)	C_27_H_47_O_12_P	10.41	[M – H]^−^	593.2745	593.2727	3.1	593, 315, 311, 277, 241, 152, 96, 78, 67
37	hexadecanoyl-sn-glycerol	C_19_H_38_O_4_	10.84	[M + H]^+^	331.2842	331.2848	–1.9	331, 313, 239, 109, 99, 95, 85, 83, 57
38	glycerol 1-stearate	C_21_H_42_O_4_	11.22	[M + H]^+^	359.3154	359.3161	–2.0	359, 267, 123, 109, 97, 95, 85, 71, 57
39	monoolein	C_21_H_40_O_4_	10.95	[M + H]^+^	357.2997	357.3005	–2.2	357, 339, 265, 247, 135, 121, 97, 95, 83, 81, 69, 57
40	panaxcerol B	C_27_H_46_O_9_	10.23	[M + HCOO]^−^	559.3128	559.3118	1.7	513, 277, 253
41	vanillin	C_8_H_8_O_3_	1.93	[M + H]^+^	153.0546	153.0552	–3.7	153, 125, 111, 97, 93, 79, 65,
42	3,4-dihydroxybenzaldehyde	C_7_H_6_O_3_	0.98	[M – H]^−^	137.0233	137.0239	–4.1	137, 119, 109, 93, 81
43	syringaldehyde	C_9_H_10_O_4_	2.45	[M + H]^+^	183.0653	183.0657	–2.3	183, 155, 140, 123, 95, 67, 55, 53
				[M – H]^−^	181.0498	181.0501	–1.5	181, 166, 151
44	carnosol	C_20_H_26_O_4_	9.08	[M + H]^+^	331.1903	331.1909	–1.9	313, 297, 289, 285, 267, 243, 225, 215, 205, 191
45	sucrose	C_12_H_22_O_11_	0.39	[M + HCOO]^−^	387.1149	387.1139	2.7	387, 341, 179, 161, 119, 113, 101, 89, 71, 59
46	pimelic acid	C_7_H_12_O_4_	1.67	[M – H]^−^	159.0654	159.0657	–2.1	159, 131, 115, 103, 97

a Maybe an artifact or solvent contaminant.

b We are aware that the ppm errors are high for a high-resolution mass spectrometer. But we decided to maintain these two compounds in the list because of the mass spectrum that matches with a high percentage of similarity; Rt = retention time in minutes without correction.

In one of the molecular families, in the negative ionization mode, six phenolic acids (1–6) were annotated, including vanillic (**1**, *m*/*z* 167.0341 [M – H]^−^), gallic (**2**, *m*/*z* 169.0134 [M – H]^−^), and protocatechuic (**3**, *m*/*z* 153.0164 [M – H]^−^). The MS^2^ spectra of these compounds predominantly show the fragment ion [M – H-44]^−^, which corresponds to the loss of the carboxyl group.

Five epicatechin gallates (7–11) were annotated, including three isomers with *m*/*z* 441.083 ([M – H]^−^), registered as epicatechin-3-*O*-gallate (**7**), epicatechin-5-*O*-gallate (**8**), and epicatechin-7-*O*-gallate (**9**). Two other isomers (**10** and **11**) with *m*/*z* 603.152 [M – H]^−^, corresponding to glycosylated epicatechin gallates, were also annotated within the same cluster. The main fragment ion in the MS^2^ spectra of these compounds ([M – H-152]^−^) corresponds to the loss of the gallate group.

A molecular family of four flavonoids/catechins (12–15) was also identified in negative ionization mode. The four nodes in this family correspond to isomers of C_15_H_14_O_6_ (*m*/*z* 289.072 [M – H]^−^) and were annotated as catechin (**12**), epicatechin (**13**), mesquitol (**14**), and iso-mesquitol (**15**). The MS^2^ fragmentation spectra of these compounds primarily show the presence of the ion *m*/*z* 245, attributed to the loss of CO_2_ from the A ring, and the ion *m*/*z* 125, resulting from cleavage in the C ring (Xu et al., 2021 ^25^). Another flavonoid, quercetin (**16**), was annotated in molecular networks without any spectral similarity to other ions (self-loop). Its fragmentation spectrum was characterized by the presence of the most intense [M – H]^−^ adduct ion, as well as the *m*/*z* 151 ion, resulting from retro-Diels–Alder fragmentation in the C ring of the flavonoid. Quercetin was also found in its glycosylated form, quercetin-3-glucoside (**17**), forming a molecular family with two nodes, including rutin (**18**). The main fragment ion in the MS^2^ spectra of these compounds (*m*/*z* 300) corresponds to the loss of the sugar moieties.^26^

Six phenylpropanoids (**19–24**) and four stilbenoids (**25–28**) were annotated in a molecular family in positive ionization mode, including sinapaldehyde (**19**, *m*/*z* 209.0809, [M + H]^+^), coniferaldehyde (**20**, *m*/*z* 179.0703, [M + H]^+^), sinapyl alcohol (**21**, *m*/*z* 193.0859, [M + H–H_2_O]^+^), and combretastatin A4 (**25**, *m*/*z* 285.1121, [M + H–H_2_O]^+^). The MS^2^ spectra of these compounds predominantly show the fragment ion [M + H-32]^+^, which is attributed to the loss of methoxyl groups in the form of methanol. Another stilbenoid, rhapontigenin (**29**, *m*/*z* = 257.0821 [M – H]^−^), was also annotated in the negative ionization mode.

In positive ionization mode, a molecular family of three furofuran lignans was also annotated as syringaresinol (**30**, *m*/*z* 419.1698, [M + H]^+^), fargesin (**31**, *m*/*z* 371.1488, [M + H]^+^), and 3-methoxy-fargesin (**32**, *m*/*z* 419.1698, [M + H]^+^). Consecutive water losses were observed in the MS^2^ spectra of these compounds, generating the ions [M + H-18]^+^ and [M + H-36]^+^. In negative ionization mode, the lignans secoisolariciresinol (**33**, *m*/*z* 361.1661 [M – H]^−^) and lariciresinol (**34**, *m*/*z* 359.1505 [M – H]) were annotated as self-loops. The fragmentation of lignan **33** produced the main fragment ion *m*/*z* 165, attributed to cleavage at the beta position, while compound **34** generated the ion [M – H-30]^−^, indicating the elimination of formaldehyde.^27^

A cluster of two glycerophospholipids was annotated in the molecular networks as 1-hexadecanoyl-sn-glycero-3-phospho-(1’-myo-inositol) (**35**, *m*/*z* 571.2899 [M – H]^−^) and 9,12,15-octadecatrienoyl-glycero-3-phospho-(1’-myo-inositol) (**36**, *m*/*z* 593.2745 [M – H]^−^). The main fragment ion in the MS^2^ spectra of these compounds ([M – H-316]^−^) corresponds to the deprotonated fatty acid portion.^28^

Three glycerides were also annotated in positive ionization mode: hexadecanoyl-sn-glycerol (**37**, *m*/*z* 331.2842, [M + H]^+^), glycerol 1-stearate (**38**, *m*/*z* 359.3154, [M + H]^+^), and monoolein (**39**, *m*/*z* 357.2997, [M + H]^+^). These compounds were characterized by the presence of fragment ions indicating the subsequent loss of CH_2_ units, as well as the ion [M + H-92]^+^, which corresponds to the elimination of glycerol.^29,30^ Additionally, panaxcerol B (**40**, *m*/*z* 559.3128), a glycosyl glyceride, was annotated in the negative ionization mode as a formate adduct ion [M + HCOO]^−^.

In addition, the GNPS library also identified three phenolic aldehydes as vanillin (**41**, *m*/*z* = 153.0546 [M + H]^+^), 3,4-dihydroxybenzaldehyde (**42**, *m*/*z* = 137.0233, [M – H]^−^), and syringaldehyde (**43**, *m*/*z* = 183.0653 [M + H]^+^, *m*/*z* = 181.0498 [M – H]^−^), with the latter observed in both ionization modes. Other compounds included the phenolic diterpene carnosol (**44**, *m*/*z* 331.1903 [M + H]^+^), sucrose (**45**, *m*/*z* 387.1149 [M + HCOO]^−^), and pimelic acid (**46**, *m*/*z* 159.0654 [M – H]^−^).

Most of the compounds annotated in this study belong to the class of phenolic compounds renowned for their antioxidant properties^30^ which are consistent with findings in samples from the *Spondias* genus, such as quercetin, rutin, gallic acid, and protocatechuic acid.^31^ Lignans have also been previously reported in the Anacardiaceae family,^32,33^ while glycerides and glycerophospholipids are commonly found in plant species.^34–36^

### *In Vivo* Toxicity

The toxicity of the optimized umbu seed extract was evaluated using the *in vivo* model with *G. mellonella* larvae by analyzing the survival rate over 168 h after administration of different concentrations of the extract, in addition to their respective controls with propylene glycol (Figure 9).

**Figure 9:**
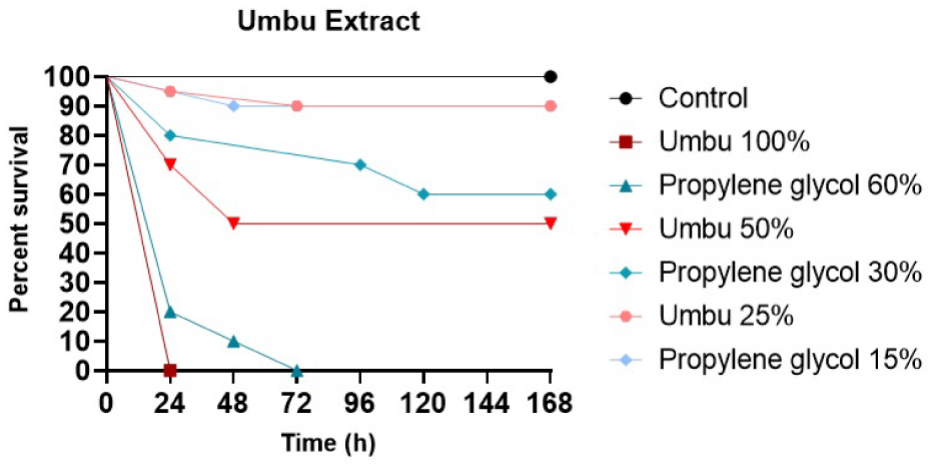
Toxicity of umbu seed extract and its corresponding solvent, propylene glycol, evaluated in *Galleria mellonella* larvae. Umbu seed extract (491 [25%] to 1963 [100%] mg GAE/L) or propylene glycol (15% to 60%).

The control group showed 100% survival throughout the experimental period, indicating no mortality due to handling. The highest dose of umbu seed extract (1,963 mg GAE/L, 100%) was highly toxic, resulting in 100% mortality in less than 24 h. The intermediate dose (982 mg GAE/L, 50%) also showed significant toxicity, with a reduction in survival to approximately 50% in the first 24 h and stabilization of this rate until the end of the experiment. In contrast, the lowest dose tested (491 mg GAE/L, 25%) did not show toxicity, maintaining a survival rate above 90%, comparable to that of the control.

Regarding the solvent, 60% propylene glycol resulted in high mortality, similar to that observed with the highest dose of the extract, indicating marked toxicity of the solvent at this concentration. The 30% propylene glycol demonstrated moderate toxicity, with a progressive reduction in survival to approximately 60% at the end of the period. The 15% propylene glycol did not present relevant toxicity, maintaining the survival rate of the larvae close to the control.

These data indicate that both the toxicity of the umbu seed extract and that of its vehicle are concentration-dependent. The total phenolic content of 491 mg GAE/L, transported in up to 15% propylene glycol, was considered safe for use in the *G. mellonella* model.

In cosmetic formulations, glycerol and propylene glycol are used as humectant agents and employed at percentages below 10%. Chamsai et al.^37^ elaborated on a plant-based sunscreen extract using 7.5% of propylene glycol in the composition. Da Silva et al.^38^ used only 0.7% propylene glycol in the preparation of a sunscreen using olive leaf extract in the composition. Thus, umbu seed extract can be safe in cosmetic formulations under the conditions tested.

### Sun Protection Factor (SPF) Assessment

According to the results obtained, shown in Figure 10, the umbu seed extract presented a photoprotective activity. It is also noted that increasing the concentration of the optimized extract from 2 mg/mL to 30 mg/mL generates a corresponding increase in the SPF, which increased from 1 to 13, respectively (*p* < 0.05). Studies such as that conducted by Mota et al.^39^ have demonstrated that, although certain plant extracts may exhibit low SPF values, their incorporation into cosmetic formulations can enhance overall photoprotective efficacy and minimize dependence on synthetic photoprotective agents.

**Figure 10:**
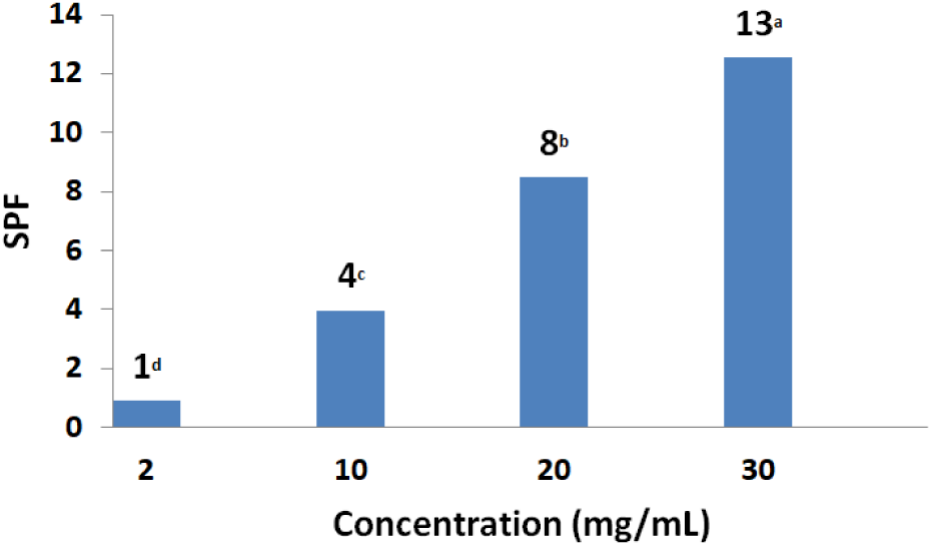
Effect of the extract concentration on sun protection factor (SPF). Bars with the same letters show that the results are equal to each other (*p* > 0.05).

## Conclusion

The extraction condition that resulted in the highest total phenolic content (TPC) and antioxidant capacity was a temperature of 201 °C, 60% propylene glycol as the solvent, and an extraction time of 15 min. Under this optimized condition, the umbu seed extract exhibited superior TPC and antioxidant capacity values compared to the extract obtained through the conventional method. The optimized extract was primarily composed of phenolic compounds including phenolic acids, catechins, flavonoids, and stilbenes. No *in vivo* toxicity was observed when 25% extract was administered to the *G. mellonella* model, with a TPC of 491 mg GAE/L in up to 15% propylene glycol. Furthermore, the extract demonstrated photoprotective activity, achieving an SPF value of 13 at a concentration of 30 mg/mL. Overall, these results demonstrate that propylene glycol is an effective green solvent for microwave-assisted extraction of umbu seed bioactives, representing a promising strategy for agroindustrial waste valorization and the sustainable use of native fruit resources. Future studies on the chemical stability of the optimized extract, its application in cosmetic formulations, and quality control of the formulated product are needed for a comprehensive evaluation of the extract’s potential.

## Materials and Methods

### Sample

The umbu seed used in this study was obtained from the depulping process carried out at the pilot plant of Embrapa Agroindústria de Alimentos, located in Guaratiba, Rio de Janeiro, Brazil. After depulping, the residual material was dried at 45 °C in a forced-air oven and then ground by using a knife mill (Wiley Mill, Philadelphia, USA) to produce a powdered sample. Granulometric analysis revealed that approximately 54% of the particles had a diameter ranging from 1.00 to 1.68 mm. The umbu seed used in this study was duly registered in the National System for Management of Genetic Heritage and Associated Traditional Knowledge (SisGen) under the number A87D2A7.

### Microwave-Assisted Solid–Liquid Extraction

To optimize the extraction of bioactive compounds from umbu seeds, an experimental design was employed, as illustrated in Table 1. The variables included propylene glycol concentrations ranging from 15% to 85% and extraction temperatures between 59 and 201 °C. The extraction time and solid–liquid ratio were fixed at 10 min and 1:30 (w/w), respectively, which conform to preliminary assays. Extractions were performed using a Milestone Ethos 1 microwave system (Sorisole, Italy) operating in a closed mode at 500–1000 W. The heating period required to reach the working temperature was set at 5 min for all experimental conditions, after which the extraction time was recorded. Following each extraction, the samples were vacuum-filtered and stored at −20 °C until analysis of total phenolic content (TPC) and antioxidant capacity. The experimental data were analyzed via response surface methodology (RSM) employing a second-order polynomial model. Analysis of variance (ANOVA), including tests for lack of fit and calculation of the coefficient of determination (*R* ^2^), was performed to evaluate the significance and adequacy of the model, with a significance level set at 5%. The desirability function was utilized to identify the optimal extraction conditions. Under these optimized conditions, additional validation experiments were conducted, and the observed results were compared with the model predictions.

### Extraction Kinetics

The kinetics evaluation was carried out by varying the extraction time from 0 to 20 min after reaching 201 °C. Propylene glycol (60%) was used as a solvent.

The temperature and percentage of propylene glycol were selected according to the optimization provided by the desirability function. At the end of the extractions, the extracts were evaluated for TPC and antioxidant capacity using the DPPH^•^, ABTS^•+^, and FRAP assays.

### Conventional Solid–Liquid Extraction

This technique was also used, in addition to microwave-assisted extraction, to compare their extraction efficiency. In this case, the extraction was performed with stirred solvent, using 50 mL glass flasks heated to 70 °C under constant stirring at 200 rpm, following the same solid–liquid ratio of 1:30 and 60% propylene glycol solvent as the optimized condition established. The extraction time was changed from 15 to 120 min to evaluate its effect on the responses. At the end of the process, the extracts obtained were filtered and stored at −20 °C until further analysis.

### Chemical Evaluation

#### Total Phenolic Content (TPC)

TPC analysis was performed using the Folin–Ciocalteu reagent according to Georgé et al.^40^ The results were obtained with the aid of a calibration curve prepared with gallic acid (Sigma-Aldrich, St. Louis, USA) solutions. TPC was expressed as milligrams of gallic acid equivalents per 100 g of sample (mg of GAE/100 g).

#### DPPH^•^ Assay

The antioxidant capacity by the DPPH^•^ assay was performed according to the methodology proposed by Hidalgo, Sánchez-Moreno, and Pascual-Teresa.^41^ Results were obtained from the elaboration of a standard curve of Trolox (Sigma-Aldrich, Buchs, Switzerland) and expressed as μmol Trolox/g of sample.

#### ABTS^•+^ Assay

The antioxidant capacity by the ABTS^•+^ assay was determined according to Gião et al.^42^ Results were obtained from the elaboration of a standard curve of Trolox (Sigma-Aldrich, Buchs, Switzerland) and expressed as μmol of Trolox/g of sample.

#### FRAP Analysis

The ferric reducing antioxidant power was performed according to Benzie and Strain.^43^ Results were calculated using a standard curve using FeSO_4_·7H_2_O (CRQ, Diadema, Brazil) solutions and expressed as μmol Fe^2+^/g of sample.

#### Ultra-High-Performance Liquid Chromatography Coupled with High-Resolution Mass Spectrometry in Tandem Analysis (UPLC-HRMS/MS)

The chemical composition of the samples (10 mg/mL in methanol) was investigated using a Thermo Dionex Ultimate 3000 Liquid Chromatograph coupled to a Thermo QExactive Plus high-resolution and accurate mass spectrometer with an electrospray ionization source operating in positive and negative ionization modes. Prior to analysis, the extract was purified through solid-phase extraction (SPE) using a Waters C18 Sep-Pak column. First, the column was conditioned by passing 10 mL of methanol and 10 mL of ultrapure water, which were discarded, and finally, 0.3 g of the sample was added to the column. After that, 15 mL of ultrapure water was eluted to obtain a propylene glycol-free extract. To recover the compounds, 10 mL of methanol and 5 mL of ethyl acetate were used.

Chromatographic separation was carried out on a Thermo Scientific Syncronis HPLC C18 column (50 mm × 2.1 mm inner diameter × 1.7 μm particle size). The mobile phase consisted of ultrapure water with 0.1% formic acid (solvent A) and methanol containing 0.1% formic acid (solvent B), using a gradient elution of 15% B (0.0–1.0 min), 15 to 95% B (1.0–8.0 min), 95% B (8.0–15.0 min), and 15% B (15.1–20.0 min). The flow rate was set at 0.35 mL/min, with a 5 μL injection volume and a column oven temperature of 40 °C. The ionization source was configured with sheath and auxiliary gases set to 45 and 15 arbitrary units, respectively. The spray voltage was set to ±3,600 V, with an S-lens voltage of 50 V. The capillary temperature was set to 300 °C, and the source temperature remained at 400 °C.

Data were acquired in full scan mode over the *m*/*z* range of 100–1,000, in positive and negative ionization modes, with a resolution of 35,000 (fwhm), an AGC of 1 × 10^6^, and an injection time (IT) of 100 ms. Data-dependent acquisition (ddMS2top3) was also performed with a resolution of 17,500 (fwhm), an AGC of 1 × 10^5^, an IT of 50 ms, a normalized collision energy (NCE) of 15–35, and an isolation window of 1.2 Da.

### Data Processing and Analysis on GNPS

The raw data obtained from the UPLC-HRMS/MS analysis were converted to the mzML format using MSConvert software (ProteoWizard Software Foundation, Palo Alto, CA, USA). The data were processed using MSDIAL software version 4.9, with mass tolerance for MS1 and MS2 set to 0.02 Da, a minimum signal intensity of 1.0 × 10^6^, and retention time (Rt) tolerance for chromatogram alignment set to 0.1 min. The processed data were submitted to the GNPS 2 platform and analyzed by using the Feature-Based Molecular Networking (FBMN) workflow. A mass tolerance of 0.02 Da was applied for both precursor and fragment ion mass tolerances, a cosine score higher than 0.70 was used, and at least 4 peaks corresponding to library spectra were required for annotation. The raw data from the molecular network generated in positive and negative ionization modes are publicly available at https://gnps2.org/status?task=f9920786b86d4818925129d0430e9316 (positive mode) and https://gnps2.org/status?task=8d2b6699902d4c36ae0f9233397af99c (negative mode).

### *In Vivo* Toxicity by the *Galleria mellonella* Model

#### Origin of Larvae

*Galleria mellonella* larvae were maintained and fed until they reached 200–300 mg in weight. The larvae were fed an artificial diet composed of honey and various flours, and the entire insect cycle was stopped at 28 °C. During the survival tests, 10 larvae were used in each study group, which were incubated at 37 °C.^44^

#### Toxicity Test

*G. mellonella* larvae weighing between 200 and 300 mg were selected for the toxicity test. Survival curves were obtained from the administration of different concentrations of umbu seed extract (491 to 1963 mg GAE/L, corresponding, respectively, to 25 to 100%) and its solvent, propylene glycol (15% to 60%). Each experimental group consisted of 10 larvae, which received 10 μL of the solution by injection using an insulin syringe, with inoculation performed in the last abdominal leg (proleg). After injection, the larvae were kept in Petri dishes and incubated at 37 °C. The control group consisted of larvae inoculated with sterile water. Survival was assessed daily for a period of 7 days (168 h), considering larvae that did not respond to tactile stimulation as dead. Statistical analysis of the survival curves was performed using the log-rank (Mantel–Cox) test, as described by Frota et al.^44^

### Sun Protection Factor (SPF) Assessment

The analysis to evaluate the SPF of the optimized extract was performed using the spectrophotometric method described by Dutra et al.^45^ Samples at different concentrations were used from 2 to 30 mg/mL. The results were obtained in triplicate in the range of 290 to 320 nm using a spectrophotometer (SP-220, Biospectro, São Paulo, Brazil). Afterward, the Mansur equation^46^ was applied to obtain the SPF values.

### Statistical Analysis

The Statistica software version 13 (Dell Inc.) was used for all statistical analysis. RSM with analysis of variance (ANOVA), Pareto chart, lack of fit, and *R* ^2^ determination were employed to verify the model significance from the experimental design, considering a confidence interval of 95%. Additionally, Pearson’s correlation was used to analyze the results of the experimental design. ANOVA and Tukey at a confidence interval of 95% were applied to the results of the kinetic evaluation and comparisons of the extraction techniques. All extraction processes were carried out in triplicate except for those conducted as part of the experimental design. Similarly, all analytical assays were performed in triplicate with the exception of the UPLC-HRMS/MS analysis.

## Supplementary Material



## Data Availability

This manuscript provides all data necessary to support the findings of the study.

## Notes

Additional details of the results obtained by UPLC-HRMS/MS are provided in Figures S1–S9 (PDF)
