# Supplementary material for: Spondias tuberosa Seed as a Source of Bioactives by an Optimized Microwave-Assisted Green Extraction
Source: ACS Omega. 2025 Nov 25;10(48):59108–20. doi: 10.1021/acsomega.5c08226 (PMC12771419; doi:10.1021/acsomega.5c08226)
Supplement: Supplementary file 1 [file ao5c08226_si_001.pdf]

Spondias tuberosa Seed as a Source of Bioactives by an Optimized Microwave-assisted Green Extraction

Ester Fonseca da Conceição[1], Carolline Margot Albanez Lorentino[2], Thayssa da Silva Ferreira Fagundes[3,4], Alex de Aguiar Novo[1], Claudete Norie Kunigami[1], André Luis Souza dos Santos[2], Davyson de Lima Moreira[3] Eliane Przytyk Jung[1] and Leilson de Oliveira Ribeiro[1]

1. Laboratory of Organic and Inorganic Chemical Analysis, National Institute of Technology, Rio de Janeiro 20081-312, Brazil;
2. Laboratory for Advanced Studies of Emerging and Resistant Microorganisms, Microbiology Institute Paulo de Góes, Federal University of Rio de Janeiro, Rio de Janeiro 21941-902, Brazil
3. Laboratory of Natural Products, Rio de Janeiro Botanical Garden Research Institute, Rio de Janeiro 22460-030, Brazil
4. Plant Biotechnology Center, Roberto Alcantara Gomes Institute of Biology, Rio de Janeiro State University. Rua São Francisco Xavier, 524, Haroldo Lisboa da Cunha Pavilion-505, Maracanã, Rio de Janeiro, 20550-013, Brazil.

*Correspondence:

leilson.oliveira@int.gov.br

Laboratory of Organic and Inorganic Chemical Analysis, National Institute of Technology, Avenida Venezuela, 82, Saúde, Rio de Janeiro 20081-312, Brazil

+552121231018 (7041)

SUPPORTING INFORMATION

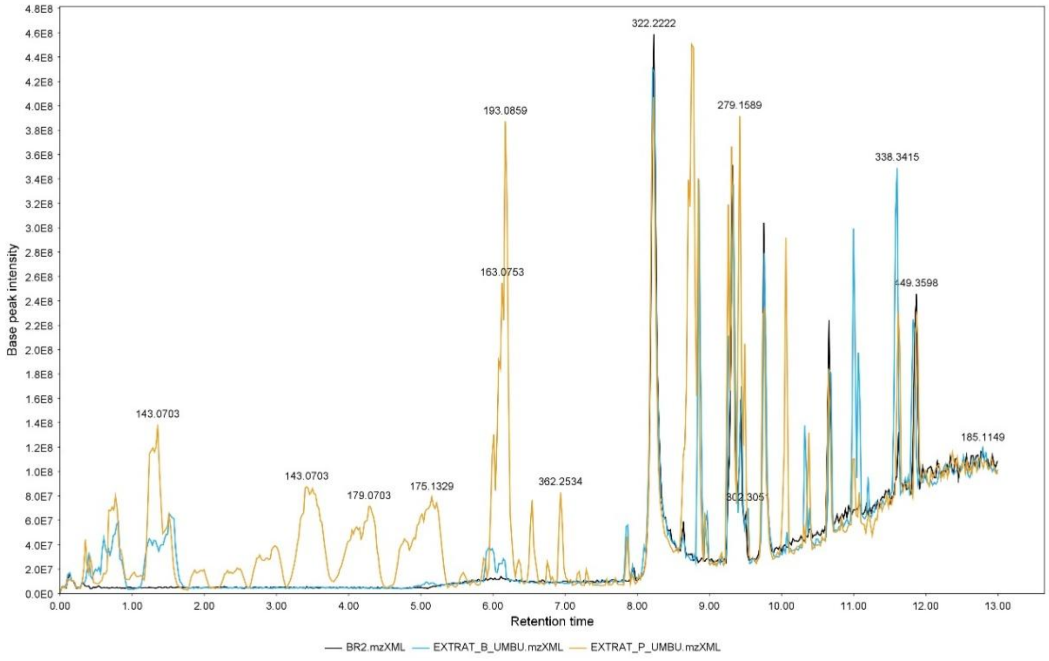

Figure S1. Overlaid chromatograms of base peak recorded (Rt 0.0 to 13.0 min) with UHPLC-HRMS/MS in positive ionization mode of the crude (UMBU B, blue color) and purified (UMBU P, orange color) extracts of *Spondias tuberosa*, in addition to the blank sample (black color).

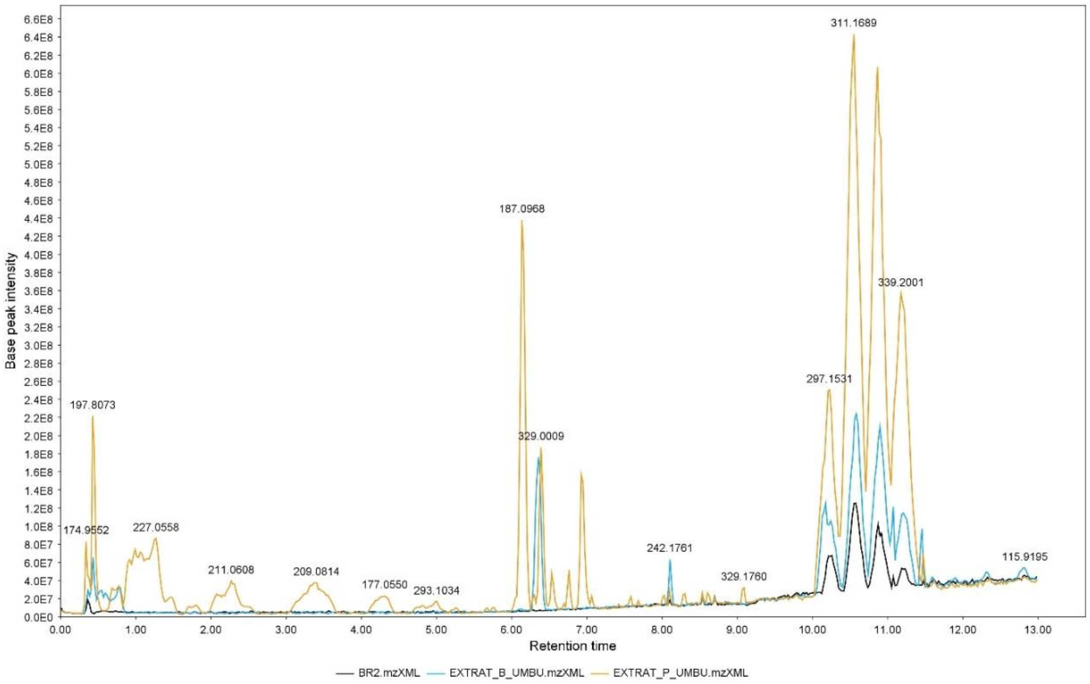

Figure S2. Overlaid chromatograms of base peak recorded (Rt 0.0 to 13.0 min) with UHPLC-HRMS/MS in negative ionization mode of the crude (UMBU B, blue color) and purified (UMBU P, orange color) extracts of *Spondias tuberosa*, in addition to the blank sample (black color)

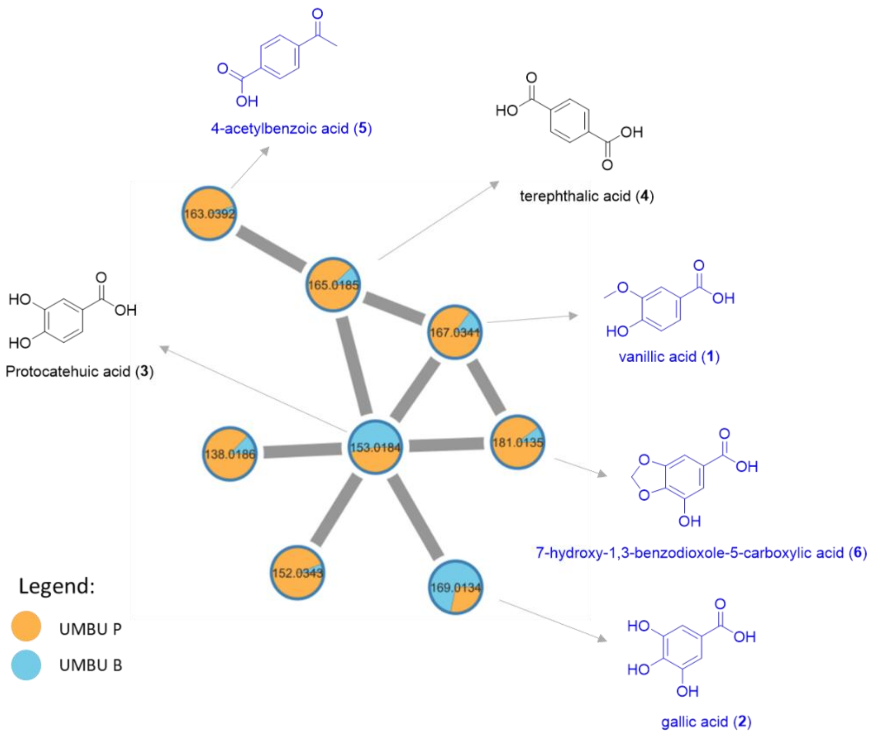

Figure S3. Phenolic acids molecular family generated by GNPS in negative ionization mode. Compounds highlighted in black color were annotated based on GNPS library matches and compounds in blue color were annotated through manual inspection and propagation of structural annotations.

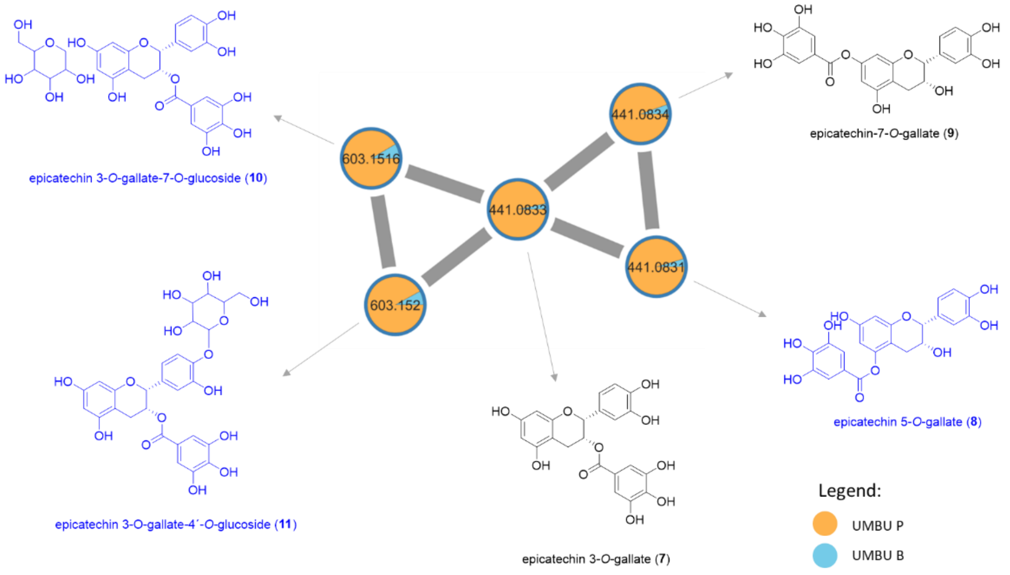

Figure S4. Catechin gallates molecular family generated by GNPS in negative ionization mode. Compounds highlighted in black color were annotated based on GNPS library matches and compounds in blue color were annotated through manual inspection and propagation of structural annotations

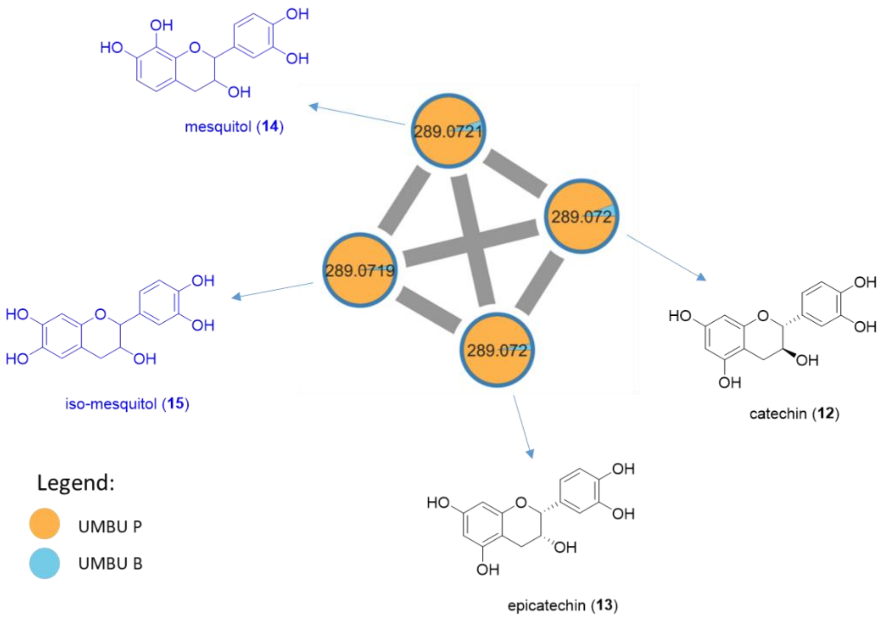

Figure S5. Catechins molecular family generated by GNPS in negative ionization mode. Compounds highlighted in black color were annotated based on GNPS library matches and compounds in blue color were annotated through manual inspection and propagation of structural annotations.

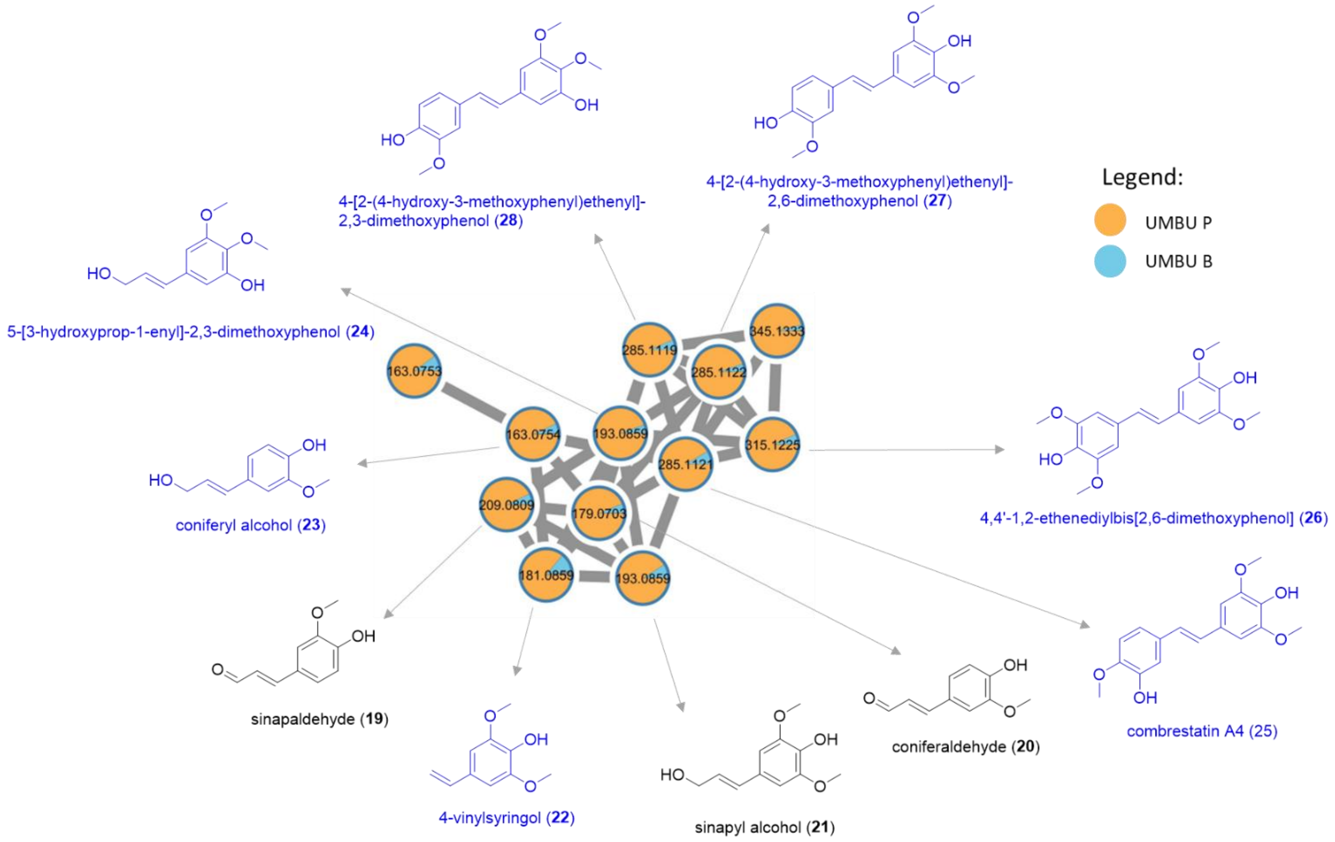

Figure S6. Phenylpropanoids and stilbenoids molecular family generated by GNPS in positive ionization mode. Compounds highlighted in black color were annotated based on GNPS library matches and compounds in blue color were annotated through manual inspection and propagation of structural annotations.

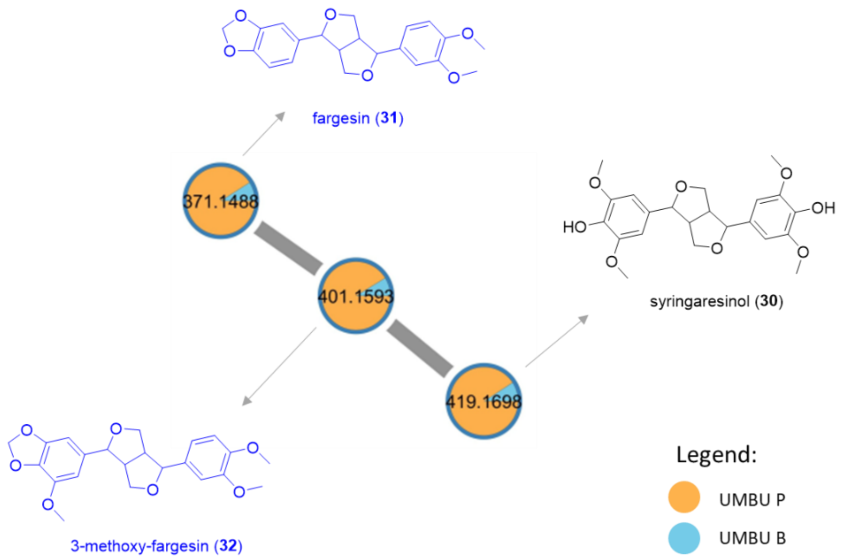

Figure S7. Lignans molecular family generated by GNPS in positive ionization mode. Compounds highlighted in black color were annotated based on GNPS library matches and compounds in blue color were annotated through manual inspection and propagation of structural annotations.

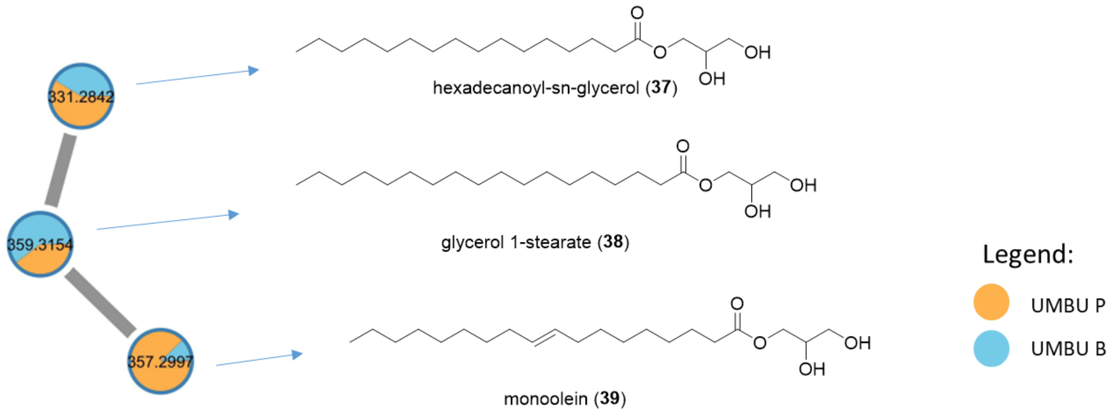

Figure S8. Glycerides molecular family generated by GNPS in positive ionization mode. Compounds highlighted in black color were annotated based on GNPS library matches and compounds in blue color were annotated through manual inspection and propagation of structural annotations.

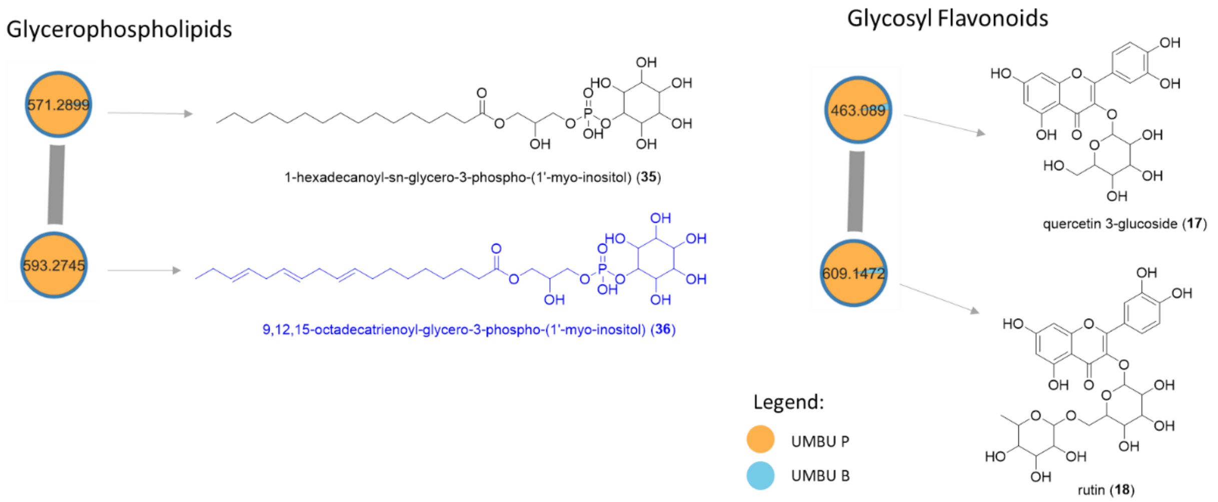

Figure S9. Glycerophospholipids and glycosyl flavonoids molecular families generated by GNPS in negative ionization mode. Compounds highlighted in black color were annotated based on GNPS library matches and compounds in blue color were annotated through manual inspection and propagation of structural annotations.
